# Influence of Vibration Modes on CaSO_4_ Scaling in Hollow-Fiber Membrane Distillation

**DOI:** 10.3390/membranes16060183

**Published:** 2026-05-27

**Authors:** Youngkyu Park, Juyoung Andrea Lee, Song Lee, Yongjun Choi, Sangho Lee

**Affiliations:** School of Civil and Environmental Engineering, Kookmin University, 77, Jeongneung-ro, Seongbuk-gu, Seoul 02707, Republic of Korea; sophia3374@kookmin.ac.kr (Y.P.); jyl20101530@gmail.com (J.A.L.); song24795@gmail.com (S.L.); choiyj1041@gmail.com (Y.C.)

**Keywords:** membrane distillation, scale formation, fouling, vibration, empirical model, patterned vibration

## Abstract

Membrane distillation (MD) is a promising technology for high-salinity water treatment, but scaling still remains a critical limitation to stable operation. This study introduces a novel approach by exploring vibration signal design as a control variable for scaling mitigation in hollow-fiber DCMD, shifting from the conventional treatment of vibration as a fixed-frequency mechanical input. The influence of different vibration modes, including fixed, random, and patterned (music-derived structured non-stationary excitation) vibrations, on CaSO_4_ scaling in hollow-fiber direct contact membrane distillation (DCMD) was systematically investigated. Bench-scale experiments were conducted using synthetic saline feed (35,000 mg/L NaCl and 2000 mg/L CaSO_4_) under both outside-in and inside-out configurations. The results reveal that vibration modifies flux decline behavior by delaying the critical volume concentration factor (*VCF_cr_*) and reducing post-critical scaling kinetics. In the outside-in mode, patterned vibration achieved the highest critical *VCF* (3.39) and lowest scale formation rate, indicating effective suppression of nucleation and crystal growth. In contrast, fixed-frequency vibration (100 Hz) was more effective in the inside-out mode, owing to resonance-induced amplification of vibration transmissibility (>140%), which enhanced local shear at the membrane surface. Spectral analysis shows that patterned vibration provides broadband and non-stationary excitation with multiple dominant frequencies, enabling continuous disruption of scaling processes, whereas random vibration lacks structured energy distribution. Furthermore, patterned vibration reduced energy consumption by 16–23% compared to fixed and random modes while maintaining comparable or superior fouling mitigation. These findings demonstrate that vibration pattern design, coupled with system resonance characteristics, is a key factor in optimizing MD performance and energy efficiency.

## 1. Introduction

Membrane distillation (MD) has emerged as a viable thermal separation technology for high-salinity water treatment, including seawater desalination, brine concentration, and industrial wastewater reuse [1,2]. Its advantages include high rejection of non-volatile solutes, operation at low hydraulic pressure, and compatibility with low-grade or waste heat sources [3,4,5,6,7,8]. These characteristics make MD particularly attractive for applications where conventional pressure-driven processes are limited by osmotic pressure. Within this framework, hollow fiber modules are gaining significant attention because they offer a much higher surface-area-to-volume ratio than flat-sheet membranes, enabling greater packing density and system compactness [9,10,11,12]. Additionally, their self-supporting structure simplifies the module assembly process and enhances industrial scalability by providing high productivity-per-unit volume while reducing the need for complex internal spacers [13,14].

Despite its potential, membrane scaling remains one of the primary barriers to stable and long-term MD operation, especially under high-recovery conditions [15]. Scaling in MD is governed by complex interactions between concentration polarization, temperature gradients, and crystallization kinetics [16,17]. As water evaporates through the membrane, solute concentration increases near the membrane surface, leading to supersaturation and subsequent crystal nucleation and growth [18]. This process typically exhibits a two-stage behavior: an initial gradual flux decline dominated by concentration effects, followed by a rapid flux collapse after reaching a critical concentration threshold [19,20]. Many studies have reported that this transition is associated with surface crystallization and pore blockage, which significantly increase mass transfer resistance and degrade performance [21,22,23,24,25]. Conventional strategies for scaling mitigation include feed pretreatment, antiscalant addition, and periodic cleaning; however, these approaches increase operational complexity and cost [21,22,23,24,25].

To address these limitations, hydrodynamic control strategies have been widely explored [26]. Enhancing shear stress at the membrane surface is known to suppress concentration polarization and reduce the residence time of supersaturated conditions [27]. Techniques such as flow turbulence promoters, spacers, and pulsatile flow have been investigated with varying degrees of success [28,29]. More recently, the mechanical vibration of membrane modules has attracted attention as an effective method to induce dynamic shear without increasing the bulk flow rate [20]. Vibration can disrupt boundary layers, promote particle detachment, and inhibit stable crystal deposition [29,30]. Previous studies have demonstrated that vibrating membranes—implemented through rotating modules, oscillating flat sheets, or vibrating hollow fibers—can significantly reduce fouling and improve flux stability [25,31,32,33]. Nonetheless, the membrane vibration approach is a comparatively novel technique in the field of MD.

It should be noted that long-term MD performance depends on the intrinsic durability of membrane materials under chemically and thermally harsh conditions. Recent advancements in selective layer highlight the multi-faceted approach required for long-term membrane performance under extreme operating environments [34]. Therefore, hydrodynamic strategies such as vibration should be considered complementary to material-based approaches: chemical robustness improves intrinsic membrane stability, whereas mechanical vibration mitigates external scaling and concentration polarization during operation.

Most existing studies, however, have focused on single-frequency (fixed) vibration, where performance is largely dependent on matching the vibration frequency with the system’s mechanical resonance [20,35]. While resonance can amplify vibration transmission and enhance fouling mitigation [36,37], it is inherently limited to narrow frequency bands and may not fully address the dynamic nature of scaling processes. In contrast, non-stationary or broadband vibration signals, such as random or patterned vibration, have received relatively little attention. These signals may provide more effective disruption of crystallization by continuously varying the excitation conditions and interacting with multiple system response modes. Despite this potential, the relationship between vibration pattern and scaling behavior remains poorly understood.

In this context, the present study investigates the influence of vibration pattern, including fixed, random, and patterned (music-derived structured non-stationary excitation) vibration, on the control of CaSO_4_ scaling in hollow-fiber DCMD. Unlike conventional approaches, this work treats vibration not merely as a mechanical input but as a signal with specific spectral characteristics, enabling a systematic comparison between fixed (resonance-driven) and patterned (broadband) vibration mechanisms.

The novelty of this study is threefold. First, unlike previous vibration-assisted membrane studies that mainly used fixed-frequency vibration, this work treats vibration as a signal-design variable and compares fixed-frequency, random, and structured non-stationary excitation for CaSO_4_ scaling control in hollow-fiber DCMD. Second, the study links vibration spectral characteristics and module transmissibility to flux decline, critical *VCF*, and empirical scaling parameters, thereby connecting mechanical excitation behavior with membrane scaling response. Third, both outside-in and inside-out configurations are examined, demonstrating that the most effective vibration strategy depends on the hydrodynamic environment of the hollow-fiber module. These features provide a new framework for designing vibration-assisted MD operation based on both spectral structure and module dynamics.

## 2. Materials and Methods

### 2.1. Membranes

Detailed properties of the MD membrane module used in this study are summarized in Table 1. The membrane is composed of polyethylene (PE) with a nominal pore size of 0.1 μm and a porosity of 70%, providing high permeability while maintaining effective vapor–liquid separation. The fiber dimensions include an inner diameter of 0.57 mm and an outer diameter of 0.83 mm, indicating a thin-wall structure that facilitates heat and mass transfer. The total effective membrane area per module is 65 cm^2^.

### 2.2. Experimental Setup for Vibration-Assisted MD

In this study, the patterned vibration was generated using a representative music-derived waveform. This waveform is hereafter interpreted not as a musical input but as a structured non-stationary excitation signal. It was selected as a practical and reproducible broadband signal containing temporally varying dominant frequencies and persistent spectral peaks. Therefore, it served as a proof-of-concept signal to examine whether structured broadband excitation can mitigate scaling more effectively than either single-frequency vibration or stochastic random vibration.

**Figure 1:**
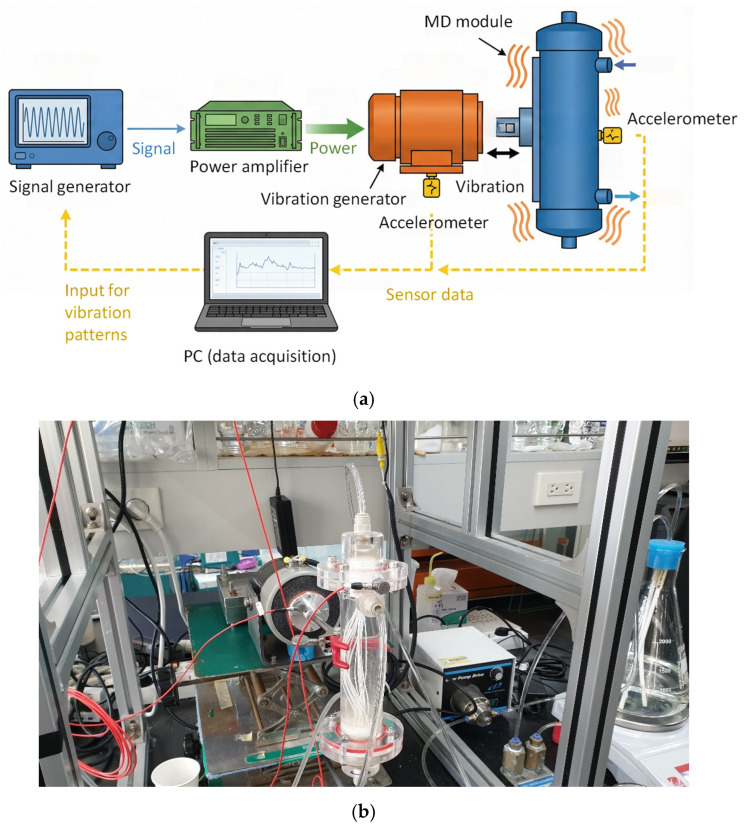
Experimental setup for vibration-assisted membrane distillation (MD). (**a**) Schematic diagram, (**b**) photography.

Figure 1a illustrates the experimental setup for vibration-assisted membrane distillation (MD). The system comprises a signal generator (m+p analyzer v5.1), a power amplifier, and a vibration generator (shaker) mechanically coupled to the MD module. The signal generator produces controlled signals, including fixed, random, and patterned waveforms, which are amplified and transmitted to the shaker to induce vibration of the membrane module. This device also allows the use of an mp3 file to create patterns for vibration applications. The signal is then amplified using the power amplifier, allowing the operation of the vibration generator. The vibration modes were compared under equal amplitude conditions. The maximum amplitude was adjusted to 1 mm for all vibration modes. The accelerometers (m+p vibpilotIEPE vibration accelerometer (m+p international, Hannover, Germany) are installed at both the vibration generator and the MD module to measure input and transmitted acceleration, respectively, enabling evaluation of vibration transmissibility. The measured signals are acquired and processed using a data acquisition system (PC) for real-time monitoring and analysis. This configuration allows precise control and characterization of vibration conditions during MD operation. The photographic image of the actual experimental setup is shown in Figure 1b.

### 2.3. Experimental Conditions

To examine the fouling behaviors of the MD membranes, the feed solution was prepared using 35,000 mg/L of NaCl and 2000 mg/L of CaSO_4_. All reagents were purchased from Sigma Aldrich (St. Louis, MO, USA). The feed flow and distillate flow rates were 0.9 L/min to 0.6 L/min with MD. The initial feed inlet temperature was 60 °C, and the distillate inlet temperature was 20 °C with MD. The frequency of vibration feed was adjusted to be continuous, random, and patterned. A representative music-derived signal was used to generate patterned broadband vibration. The experimental conditions for the MD operation are summarized in Table 2.

### 2.4. Experimental Procedures

A series of MD experiments was conducted using the feed water shown in Table 2. All the tests were performed in batch operation mode. The system was operated in both outside-in and inside-out configurations to examine the effect of flow configuration. Permeate flux was continuously monitored, and concentration was quantified using the volume concentration factor (*VCF*), defined as the ratio of the initial feed volume to the remaining feed volume during operation.

(1) $$VCF=\frac{V_{0}}{V_{0}-V_{p}}$$

where *V*_0_ is the initial quantity of feed volume, and *V_p_* is the cumulative permeate production. Experiments were performed under different vibration conditions, including no vibration, fixed-frequency (100 Hz), random, and patterned vibrations driven by a music waveform, applied continuously throughout the run. Each experiment was continued until a significant flux decline occurred, corresponding to the onset of severe scaling. The resulting flux–*VCF* profiles were used to identify the critical *VCF* and to analyze scaling behavior under different operating and vibration conditions. The experiments were repeated to confirm reproducibility, and the flux measurements were performed at least three times.

### 2.5. Analysis of Data Using an Empirical Model

A simple empirical model was introduced to compare, quantitatively, the flux decline characteristics under different vibration modes [35,38]. This model describes flux decline in MD as a combined effect of fouling and scale formation:

(2) $$R_{scale}=\left\{\begin{array}{l} k_{1}{\left(VCF-VCF_{cr},0\right)}^{n}\;\;\;\;\;\;\;VCF>VCF_{cr}\\ 0\;\;\;\;\;\;\;\;\;VCF\leq VCF_{cr} \end{array}\right.$$

(3) $$J=J_{0}e^{-k_{2}(VCF-1)}\left(\frac{1}{1+R_{scale}}\right)$$

where *R_scale_* is the relative hydraulic resistance of the scales on the membrane, *k*_1_ is the effective scale formation rate constant, *n* is the reaction order, and *VCF_cr_* is the critical *VCF*, *J*_0_ is the initial flux, and *k*_2_ is the concentration-related resistance prior to *VCF_cr_*.

The scale formation rate is defined as a threshold-dependent function of *VCF*. Below the critical *VCF*, no scaling is assumed to occur. Once the *VCF* exceeds this critical value, scaling starts and increases following a power-law relationship. The permeate flux, *J*, is then modeled as the product of two multiplicative effects. The first term accounts for the gradual flux decline prior to *VCF_cr_*. The second term represents the additional resistance imposed by scale formation after *VCF_cr_*. Together, these expressions capture the two-stage behavior observed experimentally: a mild flux decline before the critical *VCF* and a sharp decrease after scaling onset.

This empirical model was used as a comparative framework to quantify the two-stage flux-decline behavior observed during CaSO_4_-containing MD operation. The model structure reflects the experimentally observed transition from gradual pre-critical flux decline to rapid post-critical deterioration after the critical *VCF*. In this formulation, *VCF_cr_* represents the apparent onset of scaling-associated flux decline, *k*_1_ describes the apparent rate of post-critical scale-induced resistance increase, *n* represents the nonlinearity of the post-critical scaling response, and *k*_2_ accounts for gradual pre-critical resistance caused by concentration effects, viscosity increase, and boundary-layer development. Therefore, the model provides a useful basis for comparing how different vibration modes delay scaling onset and alter post-critical flux decline. However, it should be noted that the model is semi-empirical and does not explicitly resolve crystal nucleation, crystal growth, detachment, redeposition, or spatial heterogeneity of scale formation. Accordingly, the fitted parameters should be interpreted as apparent descriptors of overall scaling-associated flux decline rather than intrinsic crystallization constants.

Using the experimental data, the model parameters, *k*_1_, *k*_2_, and *n*, are determined by non-linear regression. The critical *VCF* is determined from experimental flux—*VCF* data by identifying the transition point between the two characteristic regimes of flux decline. The parameter *k*_1_ reflects the intensity of crystallization and deposition on the membrane surface after the onset of scaling. A higher *k*_1_ indicates a rapid accumulation of scale and a sharper post-critical flux decline, whereas a lower value corresponds to slower scale growth and improved resistance to fouling. The reaction order *n* describes the nonlinearity of the scaling process regarding supersaturation (represented by *VCF*-*VCF_cr_*), capturing how sensitively the scaling rate responds to increasing concentration. Larger values of *n* indicate more abrupt and unstable scaling behavior. The parameter *k*_2_ accounts for effects such as increased osmotic pressure, viscosity, and boundary layer thickening. Therefore, *k*_2_ governs the gradual flux decline in the pre-critical regime, while *k*_1_ and *n* dominate the post-critical scaling behavior.

## 3. Results and Discussion

### 3.1. Effect of Vibration Modes on MD Flux Decline

**Figure 2:**
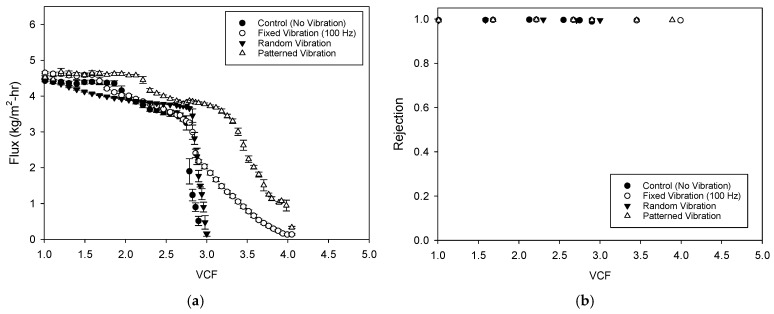
Effect of vibration on flux decline and rejection as a function of *VCF* in MD with an outside-in mode. (**a**) flux (**b**) rejection. Error bars represent the standard deviation of permeate flux obtained from repeated measurements (n_control_ = 6, n_fixed_ = 5, n_random_ = 5, n_patterned_ = 6) under identical operating conditions.

The relationship between vibration modes and flux decline as a function of *VCF* in the outside-in mode is illustrated in Figure 2. All experiments were conducted at least twice to ensure reproducibility. The variation in measured permeate flux among repeated runs was within ±10%, indicating good experimental consistency under identical operating conditions. The control condition exhibits a gradual flux decrease followed by a sharp decline at *VCF* ≈ 2.7, showing the onset of severe scaling. The salt rejection remained at a high level (99.1~99.9%) during DCMD operation, indicating that the membrane maintained effective vapor–liquid separation and that no significant pore wetting occurred. This result also aligns with previous studies on MD fouling and wetting [39]. Therefore, the observed flux decline was mainly attributed to scaling-associated resistance rather than salt leakage through the membrane. In contrast, vibration-assisted cases shift the critical *VCF* to higher values and reduce the rate of flux deterioration. Fixed vibration (100 Hz) provides moderate improvement, retarding the transition to the scaling-dominated regime to *VCF* ≈ 2.86. Random vibration shows a similar trend but with slightly enhanced stability prior to the critical point. Notably, patterned vibration achieves the most significant performance enhancement, extending the critical *VCF* to approximately 3.39 and maintaining higher flux over the entire *VCF* range. These results point out that broadband and non-stationary vibration more effectively disrupt scale formation compared to single-frequency excitation, thereby improving process stability and extending operational limits.

Direct evidence of CaSO4 scale formation and mitigation in vibration-assisted MD, including SEM observation and related membrane-surface characterization (SEM-EDX, XRD, and crystal morphology analysis), has been reported in our previous studies under comparable CaSO_4_-containing feed conditions [20,35]. Therefore, the present study did not repeat destructive post-operation surface analysis but instead focused on comparing the effects of different vibration modes using process-level indicators such as critical *VCF*, post-critical flux decline, and fitted scaling parameters. Based on previous direct characterization, the sharp flux decline observed after the critical *VCF* in this study was interpreted as CaSO_4_ scaling-associated performance deterioration.

The observed mitigation of scaling under vibration can be interpreted in terms of enhanced mass transfer and reduced concentration boundary layer thickness at the membrane surface [30,40]. In MD, the accumulation of solutes near the membrane leads to local supersaturation, which promotes nucleation and crystal growth. Vibration induces oscillatory motion in the fluid, increasing the effective mass transfer coefficient (and also the effective Sherwood number), thereby reducing the boundary layer thickness [20]. This reduction limits the buildup of supersaturated conditions and decreases the residence time for crystal nucleation. In addition, periodic shear fluctuations generated by vibration promote the detachment of nascent crystals before they can form stable deposits. As a result, both the onset of scaling is delayed, and the subsequent growth rate is suppressed, consistent with the observed increase in *VCF_cr_* and reduction in *k*_1_ under vibration conditions.

**Figure 3:**
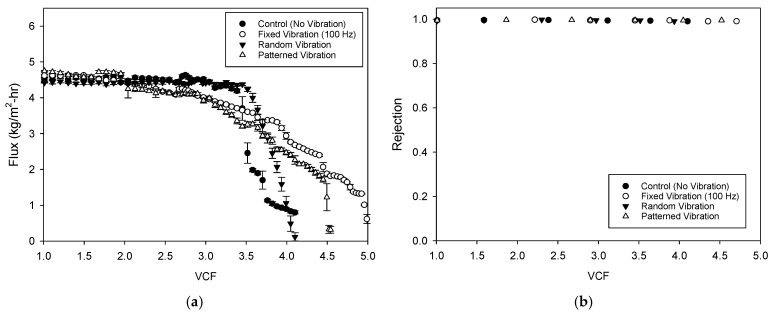
Effect of vibration on flux decline and rejection as a function of *VCF* in MD with an inside-out mode. (**a**) flux (**b**) rejection. Error bars represent the standard deviation of permeate flux obtained from repeated measurements (n_control_ = 4, n_fixed_ = 4, n_random_ = 3, n_patterned_ = 4) under identical operating conditions.

The relationship between permeate flux and *VCF* for MD operated in an inside-out mode is shown in Figure 3. Compared to the outside-in configuration, the control case shows a more gradual transition to the scaling-dominated regime but still experiences a marked flux collapse at higher *VCF*. This slower progression of scaling can be attributed to the confined lumen flow, which promotes a more uniform velocity distribution and reduces localized supersaturation near the membrane surface [41]. In addition, the internal flow geometry enhances convective transport along the fiber axis, limiting the residence time of supersaturated conditions and delaying crystal nucleation and growth. Similar to the outside-in mode, the salt rejection in the inside-out mode also remained at a high level during DCMD operation, confirming that no significant pore wetting occurred.

Vibration alters both the onset and progression of this decline. Fixed vibration (100 Hz) sustains higher flux over an extended *VCF* range, indicating delayed scaling initiation and reduced deposition rate. Random vibration further improves stability in the mid-*VCF* region, maintaining elevated flux prior to the rapid decline phase. In contrast, patterned vibration exhibits a broader operating window but with increased variability at high *VCF*, suggesting a trade-off between extended operation and instability. Overall, the results show that the effectiveness of vibration is configuration dependent. The inside-out mode primarily benefits from sustained flux retention rather than a pronounced shift in critical *VCF*.

The configuration-dependent effectiveness of vibration can be further interpreted using dimensionless hydrodynamic considerations. In the inside-out mode, the feed flows through the confined lumen of the hollow fiber. The oscillatory nature of vibration can be represented by the Womersley number, α = (D_h_/2)(ω/ν)^0.5^, where D_h_ is the hydraulic diameter, ω is the angular frequency, and ν is the kinematic viscosity [42]. Using the lumen diameter of 0.57 mm and water properties at 60 °C, α is approximately 10 at 100 Hz and ranges from approximately 4.6 to 16.3 over the frequency range of 20–250 Hz. These values indicate that oscillatory inertia is significant and that vibration creates a near-wall oscillatory shear layer rather than being fully diffused across the lumen during each cycle. Therefore, when the excitation frequency coincides with the module resonance near 100 Hz, the amplified motion can efficiently enhance local shear within the confined lumen, explaining the strong performance of fixed vibration in the inside-out mode.

In contrast, the outside-in mode involves flow over the external surface of a fiber bundle. In this configuration, a single Reynolds number or hydraulic length scale cannot fully describe the local hydrodynamics because the flow field includes inter-fiber gaps, wake regions, stagnation zones, and non-uniform external boundary layers. Consequently, the characteristic time scales for concentration polarization and crystal attachment are spatially distributed. A structured non-stationary excitation can perturb a broader range of these local hydrodynamic environments than a single fixed frequency. This explains why patterned vibration, which contains multiple dominant frequencies and time-varying spectral content, was more effective in delaying scaling onset in the outside-in mode.

**Figure 4:**
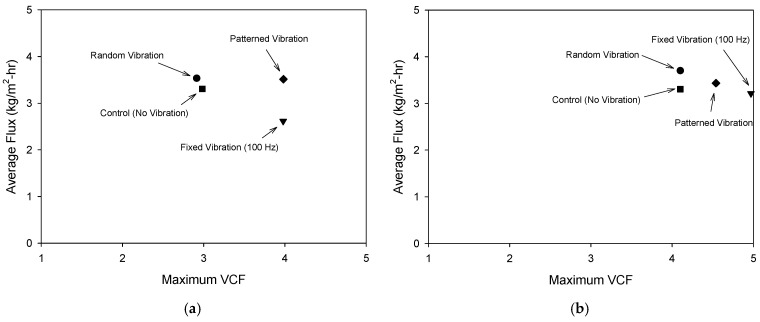
Relationship between maximum *VCF* and average permeate flux under different vibration modes in DCMD. (**a**) Outside-in mode (**b**) Inside-out mode.

The trade-off between achievable concentration (maximum *VCF*) and average permeate flux under various vibration modes is illustrated in Figure 4 for both configurations. In the outside-in mode (Figure 4a), patterned vibration attains the highest maximum *VCF* while maintaining relatively high average flux, showing effective suppression of scaling over an extended operating range. Random vibration provides a moderate improvement in flux but with a limited extension of maximum *VCF*, whereas fixed-frequency vibration shows the lowest average flux despite a comparable *VCF* to the control. In the inside-out mode (Figure 4b), fixed vibration achieves the highest maximum *VCF* with competitive average flux, suggesting enhanced tolerance to concentration buildup. Random vibration yields the highest average flux but at a slightly reduced maximum *VCF*, while patterned vibration offers balanced performance between these two metrics. These results show that the optimal vibration strategy depends on the targeted objective, such as maximizing concentration or sustaining flux, and is strongly influenced by module configuration.

**Figure 5:**
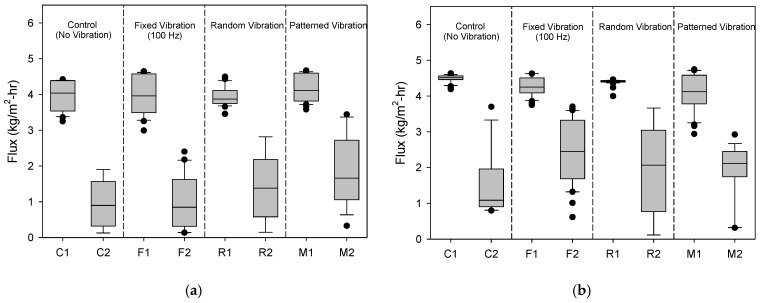
Distribution of permeate flux under different vibration modes in DCMD before and after the critical *VCF* (*VCF_cr_*). The “before *VCF_cr_*” and “after *VCF_cr_*” data were collected from the same continuous MD run without intermediate cleaning or restarting. (**a**) Outside-in mode (**b**) Inside-out mode. (C1: control before *VCF_cr_*, C2: control after *VCF_c_*_r_, F1: fixed vibration before *VCF_cr_*, F2: fixed vibration after *VCF_cr_*, R1: random vibration before *VCF_cr_*, R2: random vibration after *VCF_cr_*, M1: patterned vibration before *VCF_cr_*, M2: patterned vibration after *VCF_cr_*).

The statistical distributions of permeate flux before and after *VCF_cr_* for each vibration mode and module configuration are compared in Figure 5. The flux data before and after *VCF_cr_* were obtained from the same continuous batch MD run. No membrane cleaning, replacement, or system restart was conducted at *VCF_cr_.* The critical *VCF* was used only as a data-classification threshold to distinguish the pre-critical and post-critical flux regimes. Prior to *VCF_cr_*, all conditions exhibit narrow interquartile ranges and high median flux, showing stable operation with limited influence of scaling. After *VCF_cr_*, the control shows a substantial reduction in median flux and a substantial increase in variability, consistent with unstable operation under severe scaling. Vibration alters this distribution by both elevating the median flux and narrowing the spread. In the outside-in mode (Figure 5a), patterned and random vibrations maintain higher post-critical medians and reduce extremely low-flux outliers relative to fixed vibration and control. In the inside-out mode (Figure 5b), fixed and patterned vibrations show improved central tendency, while random vibration reduces dispersion in the high-flux regime. These shifts in distribution show that vibration not only delays scaling onset but also stabilizes flux performance after *VCF_cr_*, with the magnitude and pattern of improvement depending on vibration type and flow configuration.

### 3.2. Analysis of Flux Decline Characteristics Using an Empirical Model

**Figure 6:**
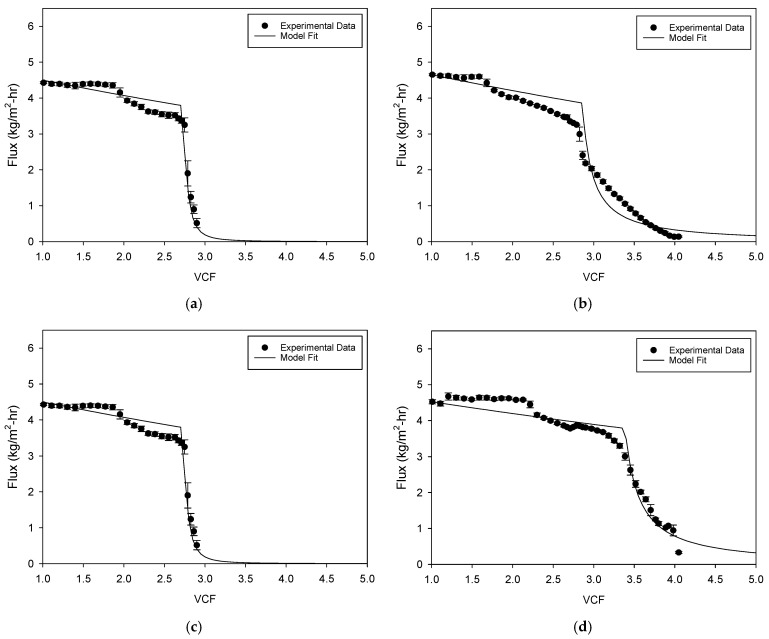
Model fit of flux decline as a function of *VCF* under different vibration conditions in MD with an outside-in mode. (**a**) control (**b**) fixed vibration (100 Hz) (**c**) random vibration (**d**) patterned vibration.

The ability of the proposed model to reproduce flux decline behavior under various vibration conditions in the outside-in configuration is evaluated in Figure 6. The model accurately captures the two-regime structure of the data, comprising a gradual pre-critical decline followed by a sharp post-critical drop, across all cases. In the control and fixed-vibration conditions (Figure 6a,b), the transition near *VCF_cr_* is well represented, although minor deviations appear immediately after the onset of rapid scaling. For random vibration (Figure 6c), the model closely follows both the slope and magnitude of flux reduction, showing consistent parameterization of scaling kinetics. In the patterned vibration case (Figure 6d), the model reproduces the extended pre-critical region and delayed transition, while slightly underestimating flux at high *VCF*, suggesting additional dynamics not fully captured by the current formulation. Overall, the agreement between experimental data and model predictions supports the validity of the proposed framework for describing vibration-modulated scaling behavior.

The impact of vibration on the model parameters governing scaling kinetics and flux decline in the outside-in configuration is quantified in Table 3. Patterned vibration exhibits the highest critical *VCF* (3.39), indicating the most effective delay of scaling onset. It also yields the lowest scale formation rate constant (*k*_1_ = 5.88) and a reduced concentration effect constant (*k*_2_ = 0.08, demonstrating simultaneous mitigation of both crystallization kinetics and concentration-induced resistance. In contrast, the control shows an earlier transition (*VCF_cr_* = 2.7) and substantially higher *k*_1_, while fixed vibration reduces *k*_1_ but provides limited improvement in *VCF_cr_*. Random vibration increases *VCF_cr_* marginally but exhibits a large *k*_1_, suggesting unstable scaling behavior. The superior performance of patterned vibration can be attributed to its broadband and non-stationary excitation, which disrupts crystal nucleation, inhibits coherent growth, and prevents stable deposition on the membrane surface. This combination enables sustained flux and extended operation beyond the critical concentration threshold.

The relatively poor performance of random vibration can be attributed to its lack of coherent frequency structure and inefficient coupling with the system dynamics. Unlike fixed or patterned vibration, random excitation distributes energy over a wide frequency range without sustained dominant components, resulting in limited resonance interaction with the membrane module. Consequently, the transmitted vibration intensity at the membrane surface remains insufficient to generate consistent shear enhancement. In addition, the irregular and intermittent nature of random perturbation may induce repeated detachment and re-deposition of loosely formed crystals, leading to unstable scaling behavior and an apparent increase in the effective scaling rate constant (*k*_1_). This explains the observed high *k*_1_ under random vibration despite only marginal improvement in *VCF_cr_*, indicating that random excitation is less effective in suppressing stable scale formation compared to structured or resonance-driven vibration. This indicates that not all broadband vibration is effective, and the presence of structured frequency components is critical for efficient fouling mitigation.

**Figure 7:**
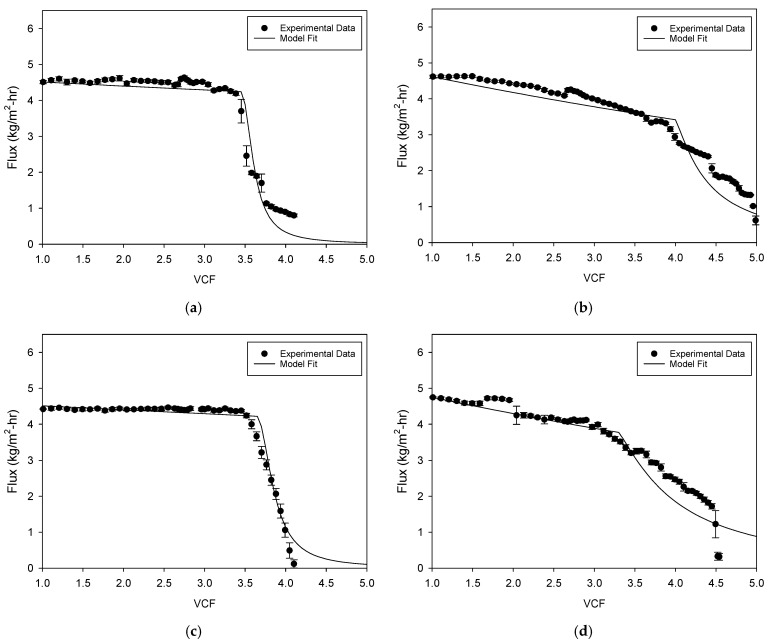
Model fit of flux decline as a function of *VCF* under different vibration conditions in MD with an inside-out mode. (**a**) control (**b**) fixed vibration (100 Hz) (**c**) random vibration (**d**) patterned vibration.

Figure 7 presents model fitting results for the inside-out configuration, emphasizing differences in post-critical behavior among vibration modes. Unlike the outside-in case, the flux decline after *VCF_cr_* is more gradual and extends over a wider *VCF* range, indicating a distributed scaling process rather than an abrupt transition. The model captures this extended decline by reproducing the continuous curvature of the flux–*VCF* relationship. The control case (Figure 7a) shows a sharper transition, consistent with localized scaling. In the patterned vibration case (Figure 7b,d), the model reflects the sustained flux at intermediate *VCF* but slightly underestimates the late-stage decline. Similar late-stage deviations between the model and experimental data were also observed in the patterned-vibration case for outside-in operation (Figure 6d). These deviations should not be interpreted as evidence of a completely different scaling mechanism. Rather, they indicate that the present semi-empirical model does not explicitly account for transient scale behaviors or vibration-induced dynamic transport. Therefore, the model is suitable for comparing overall flux-decline tendencies and fitted scaling parameters, but additional physics-based terms would be required to fully describe local transport and detachment phenomena under vibration. These results show that, in the inside-out mode, vibration changes not only the onset but also the spatial progression of scaling, requiring a more distributed representation of resistance buildup.

A distinct response to vibration is observed in the fitted model parameters for the inside-out configuration, as summarized in Table 4, which contrasts with the outside-in results. Fixed vibration yields the highest critical *VCF* (4.00) and a low scale formation rate constant (*k*_1_ = 2.92), showing the effective delay of scaling under this flow orientation. Although patterned vibration achieves the lowest *k*_1_ (1.38), its critical *VCF* (3.30) is lower than that of both fixed and random vibration, demonstrating reduced effectiveness in extending the operating limit. This contrasts with the outside-in results, where patterned vibration provided the most significant improvement in *VCF_cr_*. The reduced benefit of patterned vibration in the inside-out mode can be attributed to hydrodynamic and structural differences: in this configuration, flow-induced shear and internal channel confinement dominate mass transfer and scaling behavior, limiting the penetration and spatial uniformity of broadband vibration. As a result, the non-stationary excitation characteristic of patterned vibration is less effective in disrupting scale formation uniformly, whereas fixed-frequency vibration can more efficiently couple with the system’s dominant mechanical response and enhance local mixing.

Overall, the model reasonably captured the characteristic two-stage flux-decline behavior observed under all tested vibration conditions and enabled consistent comparison of *VCF_cr_*, *k*_1_, and *k*_2_ among operating modes. This is because flux decline generally proceeds through an initial concentration-polarization-dominated regime, followed by a rapid scaling-dominated regime after a critical concentration threshold is reached [43]. Rather than applying complex modeling approaches [38], this model provides insight into the characteristics of scaling for a given condition. This supports its use as a practical tool for evaluating vibration-assisted scaling mitigation in the present bench-scale MD system.

### 3.3. Analysis of Acceleration Transmissibility

**Figure 8:**
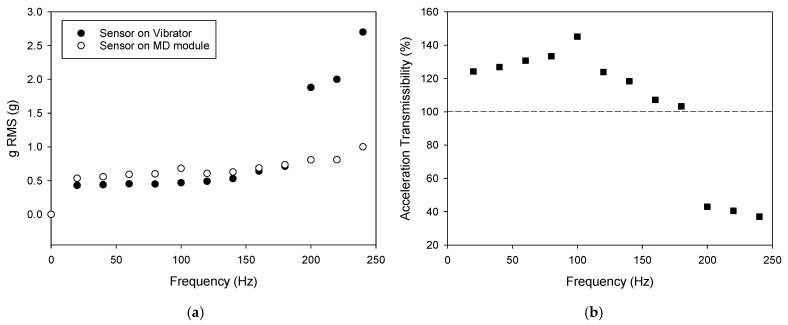
Frequency-dependent vibration characteristics and acceleration transmissibility of the MD system. (**a**) measured g RMS at two sensors (**b**) acceleration transmissibility.

To provide insight into the mechanism underlying the effectiveness of fixed-frequency vibration, the frequency response of the MD system is characterized in Figure 8. The measured g RMS (Figure 8a) increases with input frequency; however, the transmitted acceleration at the MD module does not scale linearly, reflecting structural damping and dynamic coupling. The acceleration transmissibility (Figure 8b) reaches a maximum at approximately 100 Hz, exceeding 140%, which shows resonance of the coupled vibrator–module system. At resonance, the excitation frequency coincides with the natural frequency of the structure, leading to amplification of oscillatory motion and enhanced energy transfer to the membrane module. This amplified vibration increases local shear, disrupts concentration boundary layers, and destabilizes nascent scale deposits. Consequently, fixed vibration at 100 Hz is particularly effective because it exploits this resonance condition, delivering higher effective vibration intensity to the membrane surface compared to off-resonance or broadband excitation.

### 3.4. Analysis of Frequency Patterns of Patterned Vibration

**Figure 9:**
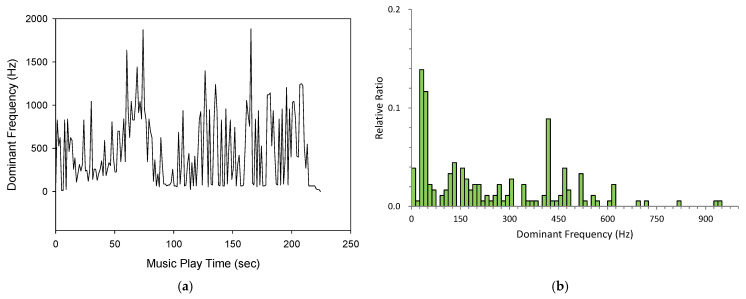
Frequency characteristics of patterned vibration used in DCMD experiments. (**a**) Temporal variation in dominant frequency during music playback (**b**) Distribution of dominant frequencies expressed as relative ratio.

To elucidate the spectral features responsible for its fouling mitigation performance, the input signal used to generate patterned vibration in the MD system is characterized in Figure 9. The dominant frequency varies continuously over time (Figure 9a), showing a non-stationary excitation rather than a fixed-frequency input. The frequency distribution (Figure 9b) confirms a broadband profile, with energy spread across low to mid frequencies and intermittent peaks at higher frequencies. This signal corresponds to the representative signal used to generate patterned vibration, and thus represents a realistic, dynamically varying vibration source. The broad spectral content enables intermittent excitation near multiple system response frequencies, promoting repeated disturbance of concentration boundary layers and scale nuclei. Consequently, patterned vibration introduces temporally and spectrally diverse perturbations that differ from single-frequency or stochastic inputs.

**Figure 10:**
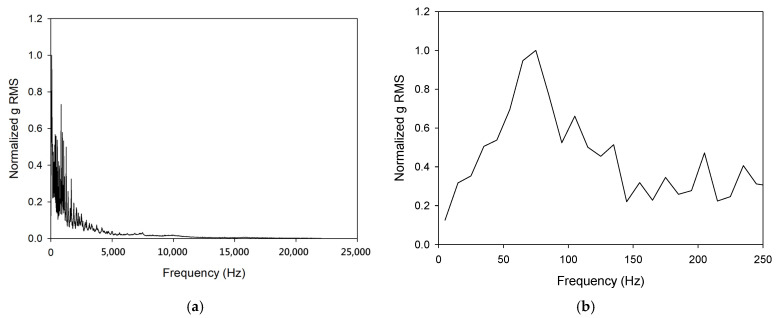
Frequency spectrum characteristics of patterned vibration applied in MD. (**a**) Normalized acceleration (g RMS) over a wide frequency range (0–25 kHz) (**b**) Enlarged view of the low-frequency region (0–250 Hz).

To provide insight into its effectiveness for scaling mitigation, the spectral energy distribution of the patterned vibration is quantified in Figure 10. The normalized g RMS spectrum (Figure 10a) shows that vibration energy is concentrated in the low-frequency range, while the enlarged view (Figure 10b) reveals distinct peaks distributed across multiple frequencies below 250 Hz. These frequencies coincide with the range where hydrodynamic boundary layer disruption and particle detachment are most effective. The presence of multiple dominant peaks enables repeated excitation of different system response modes, enhancing shear fluctuations at the membrane surface. In contrast to random vibration, which distributes energy more uniformly and lacks persistent dominant frequencies, patterned vibration combines broadband characteristics with structured spectral peaks. This hybrid behavior allows both continuous perturbation and intermittent resonance-like amplification, leading to more effective disruption of scale nucleation and growth. 

The enhanced performance of the patterned vibration should not be attributed to the musical content itself. Rather, the signal should be understood as a structured non-stationary excitation with broadband spectral density and multiple persistent dominant peaks [44,45]. Compared with random or white-noise-like excitation, which distributes energy over a wide frequency range without stable spectral features, the patterned signal retained identifiable frequency components in the low-frequency range. These components can intermittently interact with the mechanical response of the MD module, including the resonance region around 100 Hz, while also providing continuous temporal perturbation of the concentration boundary layer. Thus, the present results suggest that structured spectral density, rather than randomness alone, is important for efficient coupling between vibration input, module dynamics, and scaling mitigation.

### 3.5. Energy Consumption of Vibration Generation by Frequency

To evaluate the energy efficiency of vibration-assisted MD, the energy consumption associated with vibration generation was quantified using a digital power meter for each operating condition. Table 5 compares the energy consumption of different vibration modes applied in the MD system. In addition to the measured power consumption, the energy consumption was normalized by the maximum *VCF* obtained under each operating condition. This normalized value provides a practical index of energy requirement relative to the achievable concentration limit, because maximum *VCF* directly reflects the ability of each vibration mode to delay severe scaling. For fixed-frequency vibration, power demand increased from 39 W at 20 Hz to approximately 60–61 W above 180 Hz. Under the 100 Hz condition used in the MD experiments, the normalized energy consumption was 13.3 W for the outside-in mode and 10.7 W for the inside-out mode, indicating that fixed vibration was more efficiently utilized in the inside-out configuration, likely due to resonance-enhanced transmissibility. Random vibration required 56 W and showed the highest normalized energy consumption, 18.8 W for outside-in and 13.7 W for inside-out, indicating that its relatively high power demand did not lead to a proportional improvement in scaling mitigation. In contrast, patterned vibration consumed only 47 W and exhibited the lowest normalized energy consumption in the outside-in mode, 11.8 W, as well as a low value in the inside-out mode, 10.4 W. These results suggest that patterned vibration provided a favorable balance between power input and scaling mitigation, especially in the outside-in configuration.

Considering both power demand and flux–*VCF* behavior, patterned vibration showed a favorable balance between energy input and scaling mitigation under the tested conditions. The patterned signal consumed 47 W, which was lower than random vibration and high-frequency fixed vibration, while maintaining stable flux and extending the operating *VCF*, particularly in the outside-in mode. This result suggests that the appropriate design of the temporal and spectral structure of vibration can improve the efficiency of vibration-assisted scaling control. However, vibration intensity remains an important operating parameter, and increasing intensity can also enhance shear and fouling/scaling control to some extent. Therefore, the present results do not imply that signal design should replace intensity optimization; rather, they indicate that both spectral structure and vibration amplitude should be optimized together for energy-efficient MD operation.

### 3.6. Limitations of This Study and Future Works

This study demonstrates the potential of vibration-assisted DCMD, particularly patterned (music-derived) vibration, for mitigating scaling. However, several limitations should be acknowledged. The proposed model is a semi-empirical formulation that captures two-stage flux decline but does not explicitly resolve nucleation, crystal growth, detachment, or spatial heterogeneity. In addition, vibration was characterized mainly using frequency-domain metrics, while key factors such as amplitude distribution, phase behavior, and fluid–structure interaction were not directly quantified. The anti-scaling mechanism of patterned vibration also requires further investigation. Experimentally, only one music source and one fixed frequency (100 Hz) were tested, and the study was conducted under a single feed composition and module configuration, limiting generalizability.

Future work should develop physics-informed models coupling crystallization kinetics with transport under dynamic vibration, supported by in situ diagnostics (e.g., optical coherence tomography) to resolve spatiotemporal scaling behavior. Systematic studies on vibration signal design, including frequency, amplitude, and waveform, are needed, along with broader operating conditions and feed chemistries. Scale-up and long-term validation should also be performed to assess practical feasibility. Although this study is limited, it demonstrates the potential of patterned vibration tailored to specific membrane processes and fouling characteristics as an effective and energy-efficient scaling mitigation strategy.

The present study only used a music-derived signal as a representative structured non-stationary excitation. Other synthetic broadband signals, including chirp, swept-sine, frequency-modulated, and multi-tone waveforms, should be systematically compared in future work. Such studies would allow the spectral density, amplitude distribution, and temporal structure of the excitation signal to be independently optimized.

Moreover, the possible mechanical impact of continuous vibration on hollow-fiber integrity should also be considered in future work. Although resonance-enhanced vibration can improve local shear and scaling mitigation, excessive or prolonged operation near resonance may increase cyclic stress on the fiber wall, potting region, and module housing. Such stress could potentially induce fiber fatigue, pore deformation, or changes in wetting resistance during extended operation. Therefore, the vibration intensity and frequency should be optimized by balancing hydrodynamic benefits with mechanical safety. Because the present study focused on scaling mitigation over a defined *VCF* range, long-term cyclic fatigue tests and post-operation structural characterization were outside the present scope. Future studies should include SEM observation, liquid entry pressure measurements, pore-size distribution analysis, tensile/fatigue testing, and long-duration operation to verify whether vibration-assisted MD can be applied without compromising membrane integrity.

The present study was conducted using a bench-scale hollow-fiber DCMD module under a limited set of operating conditions, including one feed composition, one membrane type, and one representative structured non-stationary signal. Therefore, the results should not be generalized directly to other membrane materials, module geometries, feed chemistries, or long-term industrial operation without further validation. The findings should be interpreted as evidence that vibration signal design can influence scaling behavior under the tested conditions, rather than as a universal optimization rule for all MD systems.

## 4. Conclusions

This study systematically evaluated the influence of vibration patterns on CaSO_4_ scaling in hollow-fiber DCMD and demonstrated that both the spectral characteristics and system-level coupling of vibration govern fouling mitigation performance.

(1) Vibration influences flux decline by delaying scaling onset (increasing *VCF_cr_*) and reducing post-critical scaling kinetics (*k*_1_). Patterned vibration was most effective in the outside-in mode, whereas fixed vibration (100 Hz) performed better in the inside-out mode due to resonance effects under confined flow.
(2) Vibration effectiveness depends on system dynamics. Fixed vibration at 100 Hz matches the resonance frequency, maximizing transmissibility (>140%) and local shear, while patterned vibration provides broadband excitation that induces repeated perturbations across multiple frequencies.
(3) Patterned vibration is the most energy-efficient, achieving comparable or improved scaling mitigation with 16–23% lower energy consumption than fixed and random modes.

Overall, the results suggest that vibration signal design can be an important factor influencing CaSO_4_ scaling behavior in hollow-fiber DCMD under the tested bench-scale conditions. Patterned vibration was beneficial in the outside-in configuration, whereas fixed-frequency vibration was more favorable in the inside-out configuration, likely due to resonance-enhanced transmissibility. Since this study was limited to lab-scale, further long-term and scale-up studies are required to verify the practical applicability of this approach under broader operating conditions.

## Figures and Tables

**Table 1:** Automatic-threshold segmentation results for fouling area measurements.

Parameters	Conditions
Membrane Material	PE (polyethylene)
Fiber inside diameter (mm)	0.57
Fiber outside diameter (mm)	0.83
Pore size (µm)	0.1
Porosity (%)	70
Membrane area per module(cm^2^)	65

**Table 2:** Experimental Conditions for MD experiments.

Parameters	Conditions
Raw water	NaCl 35,000 ppm, CaSO_4_ 2000 ppm
Feed flow rate	0.9 L/min
Distillate flow rate	0.6 L/min
Feed inlet temperature	60 °C
Distillate inlet temperature	20 °C
Module operation mode	Outside-in/Inside-Out
Vibration mode	Fixed (100 Hz)
	Random
	Pattern (Music-derived)

**Table 3:** Estimated model parameters for flux decline and scaling behavior in MD with an outside-in mode under different vibration conditions.

Parameters	Control (No Vibration)	Fixed Vibration	Random Vibration	Patterned Vibration
Critical *VCF*	2.7	2.86	2.85	3.39
Scale formation rate constant (*k*_1_)	207.12	8.39	1046.45	5.88
Scale formation reaction order (*n*)	2.00	1.00	2.00	1.00
Concentration effect constant (*k*_2_)	0.10	0.10	0.10	0.08

**Table 4:** Estimated model parameters for flux decline and scaling behavior in MD with an inside-out mode under different vibration conditions.

Parameters	Control (No Vibration)	Fixed Vibration	Random Vibration	Patterned Vibration
Critical *VCF*	3.45	4.00	3.64	3.30
Scale formation rate constant (*k*_1_)	38.39	2.92	20.89	1.38
Scale formation reaction order (*n*)	2.00	1.20	2.00	1.20
Concentration effect constant (*k*_2_)	0.03	0.10	0.03	0.10

**Table 5:** Energy consumption of vibration modes applied in MD experiments.

Vibration Type	Frequency	Energy Consumption (W)	Energy Consumption (W) Normalized by Maximum *VCF*	
			Outside-In	Inside-Out
Fixed frequency vibration	20	39		
	40	41		
	60	45		
	80	49		
	100	53	13.3	10.7
	120	56		
	140	59		
	160	59		
	180	60		
	200	60		
	220	60		
	240	61		
Random		56	18.8	13.7
Patterned vibration		47	11.8	10.4

## Data Availability

The data presented in this study are available on request from the corresponding author. The data are not publicly available due to ongoing related research and further analysis.

## Abbreviations

The following abbreviations are used in this manuscript:

MD	Membrane Distillation
DCMD	Direct Contact Membrane Distillation
*VCF*	Volume Concentration Factor
*VCF_cr_*	Critical Volume Concentration Factor
PE	Polyethylene
g RMS	Root Mean Square Acceleration
FFT	Fast Fourier Transform
*k*_1_	Scale Formation Rate Constant
*k*_2_	Concentration Effect Constant
*n*	Reaction Order (Scaling Kinetics)
